# A structure-based rationale for sialic acid independent host-cell entry of Sosuga virus

**DOI:** 10.1073/pnas.1906717116

**Published:** 2019-10-07

**Authors:** Alice J. Stelfox, Thomas A. Bowden

**Affiliations:** ^a^Division of Structural Biology, Wellcome Centre for Human Genetics, University of Oxford, OX3 7BN Oxford, United Kingdom

**Keywords:** virus–host interaction, viral attachment, paramyxovirus, structure, glycoprotein

## Abstract

The bat-borne paramyxovirus, Sosuga virus (SosV), is one of many paramyxoviruses recently identified and classified within the newly established genus *Pararubulavirus*, family *Paramyxoviridae*. The envelope surface of SosV presents a receptor-binding protein (RBP), SosV-RBP, which facilitates host-cell attachment and entry. Unlike closely related hemagglutinin neuraminidase RBPs from other genera of the *Paramyxoviridae*, SosV-RBP and other pararubulavirus RBPs lack many of the stringently conserved residues required for sialic acid recognition and hydrolysis. We determined the crystal structure of the globular head region of SosV-RBP, revealing that while the glycoprotein presents a classical paramyxoviral six-bladed β-propeller fold and structurally classifies in close proximity to paramyxoviral RBPs with hemagglutinin-neuraminidase (HN) functionality, it presents a receptor-binding face incongruent with sialic acid recognition. Hemadsorption and neuraminidase activity analysis confirms the limited capacity of SosV-RBP to interact with sialic acid in vitro and indicates that SosV-RBP undergoes a nonclassical route of host-cell entry. The close overall structural conservation of SosV-RBP with other classical HN RBPs supports a model by which pararubulaviruses only recently diverged from sialic acid binding functionality.

## Significance

Bat populations constitute a reservoir for numerous viruses with human and animal spillover potential. Sosuga virus (SosV), from the genus *Pararubulavirus*, family *Paramyxoviridae*, is a prominent example as it has been implicated to be responsible for severe disease in an infected patient. Through investigation of the virion envelope-displayed SosV host-cell receptor binding protein, we provide a molecular-level rationale for how SosV undergoes a sialic acid-independent host-cell entry pathway, which contrasts the glycan reliance of related orthorubulaviruses, including mumps virus. By analogy to glycan-independent host-cell attachment of pathogenic henipaviruses, these data support a model whereby the evolutionary departure of SosV and other pararubulaviruses from a sialic acid-specific ancestral paramyxovirus may contribute to the extensive known host range of these emerging pathogens.

The emergence of pathogenic paramyxoviruses, such as Nipah virus (NiV) and Hendra virus (HeV) (1), from native host reservoirs into human populations has driven recent efforts to survey virus prevalence in animals. Such investigations, especially those performed in bat populations (2–7), have revealed unexpected genomic breadth, expanding the number of genera within the *Paramyxoviridae* family (8, 9) and highlighting the potential for novel paramyxoviruses to spill over and threaten human health and animal husbandry.

In 2012, a wildlife biologist was infected with one such novel paramyxovirus, Sosuga virus (SosV), following work with rodents and bats in South Sudan and Uganda (4, 10). Although not fatal, clinical manifestations of disease were severe and included acute febrile illness. Further investigation revealed that SosV may use Egyptian rousette bats (*Rousettus aegyptiacus*) as a natural host reservoir and is most closely related to the rubula-like paramyxovirus, Tuhoko virus 3 (5, 11). Along with seven other paramyxoviruses, many of which have been demonstrated to be capable of infecting humans and domestic animals (5, 12–15), SosV classifies into the newly created genus, *Pararubulavirus* (8, 9). Furthermore, recent studies of paramyxovirus circulation in South African Egyptian rousette bat populations has led to the discovery of numerous other pararubulaviruses, raising concerns about the widespread range of these putatively functionally similar viruses (6). The potential health threat posed by SosV and other pararubulaviruses has motivated the development of assays capable of screening SosV distribution and prevalence in bat populations and at-risk communities (6, 10).

The specific interaction between a paramyxovirus receptor-binding protein (RBP) and host-cell surface receptor during host-cell entry is a primary determinant of cellular and species tropism (16, 17). As type II integral membrane proteins, paramyxoviral RBPs consist of an N-terminal cytoplasmic region, transmembrane domain, stalk region, and C-terminal six-bladed β-propeller receptor-binding domain. Paramyxoviral RBPs organize as dimer-of-dimers on the viral envelope, with the receptor-binding heads forming dimers and the stalk regions driving tetramization through disulphide bonding (18–24). Paramyxoviral RBPs functionally categorize into three groups: hemagglutinin-neuraminidase (HN), hemagglutinin (H), and glycoprotein (G) (25). Unlike HN RBPs, which recognize and hydrolyze sialic acid presented on host cells, H and G RBPs attach to proteinous receptors, such as SLAMF_1_ (26–28) and ephrin receptors (29, 30), respectively. Recognition of a host-cell surface receptor by the C-terminal β-propeller domain of the paramyxoviral RBP is thought to induce allosteric rearrangements to the stalk region, which prompt the associated fusion glycoprotein to catalyze merger of the viral and host-cell membranes (31–34).

Residues responsible for hydrolysis and release of *N*-acetylneuraminic acid (Neu5Ac) by the paramyxoviral HN RBP are evolutionarily conserved and found in sialidases of eukaryotes, prokaryotes, and other virus families, indicative of a conserved mechanism of action (35–38). The sialidase catalytic site typically consists of seven residues Arg_1_, Asp_1_, Glu_4_, Arg_4_, Arg_5_, Tyr_6_, and Glu_6_ (subscript refers to location on blades 1−6 of the β-propeller fold). The arginine triad (Arg_1_, Arg_4_, and Arg_5_) binds the carboxyl group of sialic acid, Tyr_6_ and Glu_4_ form the floor of the active site, Asp_1_ is positioned over the glycosidic oxygen, and Glu_6_ stabilizes Arg_1_ (35–38). Site-directed mutagenesis of the individual residues within this conserved site has been shown to result in ablation of enzymatic acitivity (38, 39). Paramyxoviruses also express an “Asn−Arg−Lys−Ser−Cys−Ser” hexapeptide motif, a stretch of amino acids conserved among most paramyxoviral HN RBPs. Mutagenesis studies of the HN RBP from Newcastle disease virus (NDV) revealed that the first four residues of this motif (Asn−Arg−Lys−Ser) are essential for neuraminidase activity (40). Furthermore, NDV-RBP has been shown to present a second sialic acid binding site, which is located at the homodimer interface and implicated in maintaining avidity during the fusion process (41, 42).

Pararubulaviruses are closely related to orthorubulaviruses such as mumps virus (MuV), which encode RBPs with HN functionality. However, despite this close genetic relationship, a recent study revealed that pararubulaviruses likely utilize a sialic acid independent mode of entry (7), a finding rationalized by the lack of the conserved amino acid sequence required for binding and hydrolyzing sialic acid. Here, through analysis of the RBP from SosV, an emerging member of the *Pararubulavirus* genus associated with human infection, we provide an integrated structural and functional rationale for how pararubulaviruses undergo sialic acid-independent host-cell entry and egress. These data demonstrate the pathobiological distinctiveness of pararubulaviruses and highlight the diverse host-cell entry pathways available to paramyxoviruses more generally.

## Results

### SosV-RBP Lacks Hemadsorption and Neuraminidase Activity.

**Fig. 1.**
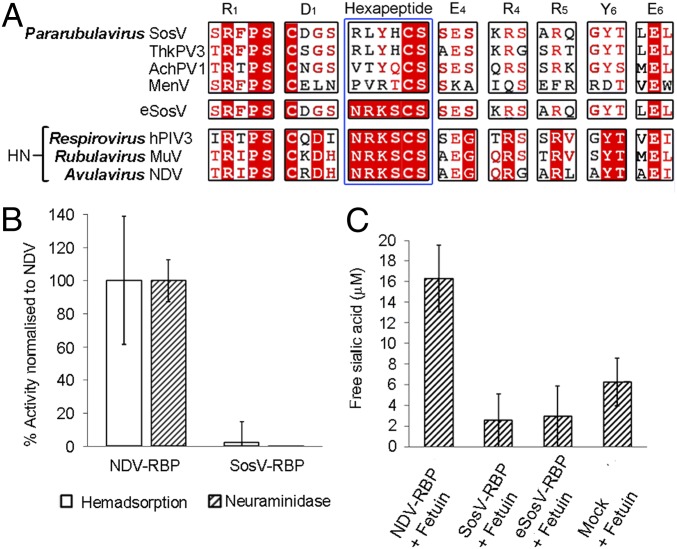
Amino acid sequence alignment and functional analysis indicate that SosV likely uses a sialic acid-independent mode of entry. (*A*) Aligment of the RBP amino acid sequences from SosV (YP_009094033.1), Tuhokovirus 3 (ThkPV-3) (YP_009094079.1), Achimota virus 1 (AchPV1) (YP_009094457.1), Menangle virus (MenV) (AAK62284.1), human parainfluenza virus 3 (hPIV3) (AAP35240.1), mumps virus (MuV) (BAA76983.1), Newcastle disease virus (NDV) (Q9Q2W5.1), and a construct of SosV engineered to incorporate the full hexapeptide motif (termed eSosV). The seven conserved sialidase residues (35, 37) and hexapeptide motif (40) are labeled according to residue and blade location (35) and annotated above the alignments. (*B*) SosV-RBP neuraminidase (48) and hemadsorption (47) activity normalized to cell surface expression and a NDV-RBP control. (*C*) Free sialic acid concentration detected following incubation of NDV-RBP, SosV-RBP, “NRKS” mutant eSosV-RBP, and mock-transfected cell supernatant with fetuin (49). For *B* (*n* = 10) and *C* (*n* = 6), error bars represent the SD.

The RBPs of SosV and other pararubulaviruses exhibit the highest level of sequence conservation with the RBPs of orthorubulaviruses (e.g., MuV-RBP) (10), a group of viruses with HN activity (43). Interestingly, while the RBP of SosV and other pararubulaviruses retain all seven residues of the sialidase catalytic site, which are conserved among the sialidase protein family more widely (35–38), the glycoproteins retain only the two C-terminal amino acids (Cys−Ser) of the hexapeptide motif known to be necessary for paramyxovirus RBP HN functionality (Fig. 1*A*). The absence of these crucial residues has also been observed in other recently classified parabulaviruses, including Menangle virus (MenV), Teviot virus (TevPV), and Tioman virus (TioV), with experimental data confirming that sialic acid is not integral to infection of permissive cells (7, 44–46).

We performed hemadsorption (47) and neuraminidase activity (48) assays to assess whether the absence of the hexapeptide motif found in HN RBPs impairs the ability of SosV-RBP to bind and hydrolyze sialic acid. In line with previous studies, which demonstrate that disruption of this key motif in NDV-RBP compromises neuraminidase activity (40), human embryonic kidney (HEK) 293T cells presenting full-length SosV-RBP exhibited no detectable neuraminidase and minimal hemadsorption functionality (Fig. 1*B*) when compared to a WT NDV-RBP control. The absence of SosV RBP neuraminidase functionality, in respect to an NDV-HN control, was further confirmed by the Warren method (49), where the concentration of free sialic acid was measured following incubation of SosV-RBP with the heavily sialylated protein, fetuin, which presents both α2,3-linked and α2,6-linked sialic acid (Fig. 1*C*) (50–53). To assess whether the introduction of the residues missing from the hexapeptide motif would enable SosV to interact with sialic acid, we compared the neuraminidase activity of a recombinantly engineered SosV-RBP (termed “eSosV-RBP”) bearing the full hexapeptide motif “Asn−Arg−Lys−Ser−Cys−Ser” (Fig. 1*A*) with WT SosV-RBP and control NDV-RBP. Similar to WT SosV-RBP, eSosV-RBP was properly folded yet exhibited no neuraminidase activity with respect to the NDV-HN positive control and a mock-transfected negative control (Fig. 1*C* and *SI Appendix*, Fig. S1), supportive of the hypothesis that the local environment surrounding the hexapeptide motif also plays a role in supporting HN functionality.

### The Structure of SosV-RBP Is Most Closely Related to Paramyxoviral HN Glycoproteins.

**Fig. 2.**
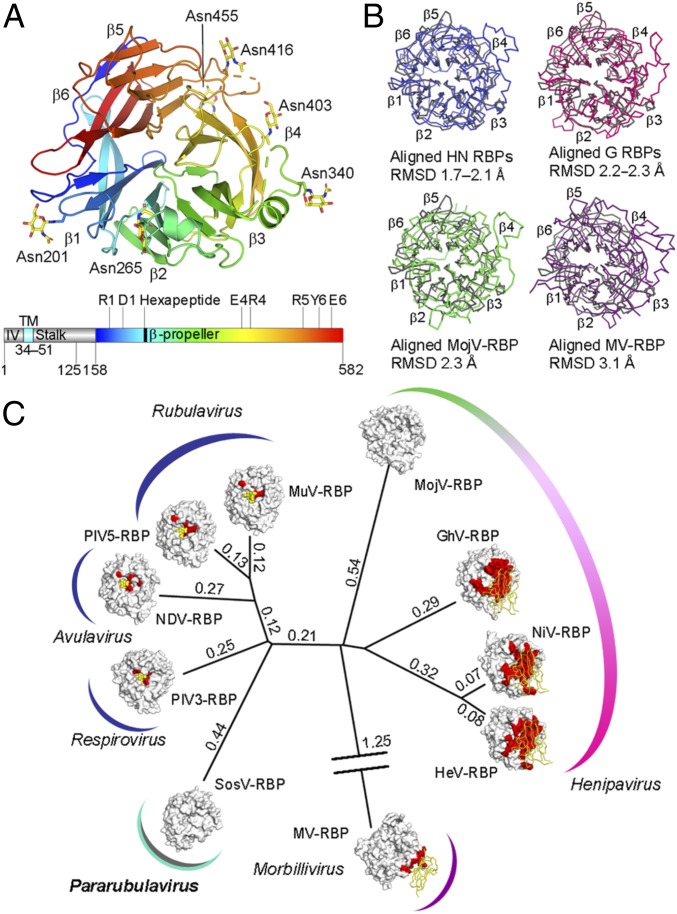
Structural relationship of SosV-RBP with other paramyxoviral RBPs. (*A*) Structure of a SosV-RBP β-propeller domain with propellers labeled (colored blue to red from N to C terminus, cartoon representation). Crystallographically observed N-linked glycosylation is represented as yellow sticks. Gene diagram with the predicted intraviral region (IV), transmembrane domain (TM), stalk, and β-propeller region annotated (colored as above). The sialidase residues and hexapeptide motif are annotated. (*B*) Overlays of SosV-RBP (gray) with other paramyxoviral RBP structures: clockwise from *Upper Left*, NDV, Newcastle disease virus (blue, 1E8V) (36); NiV, Nipah virus (pink, 2VWD) (94); MV, measles virus (2ZB5) (21); MojV, Mojiang virus (green, 5NOP) (62). Cα trace rendered and RMSD annotated. (*C*) Structure-based phylogenetic analysis of paramyxoviral RBP monomers: SosV, Sosuga virus; PIV3, parainfluenzavirus 3 (1V2I) (63); NDV (1E8V) (36); PIV5, parainfluenza virus 5 (4JF7) (64); MuV, mumps virus (5B2C) (43); MojV (5NOP) (62); GhV, Ghana virus (4UF7) (61); NiV (2VWD) (94); HeV, Hendra virus (2X9M) (22); MV (2ZB5) (21). Evolutionary distance matrices were calculated through pairwise superposition of RBP structures using SHP (60), and the unrooted tree was plotted in PHYLIP (92). RBPs are shown with surface representation. Relevant receptors are represented using ribbon (protein) or sphere (carbohydrate) (yellow). Known receptor binding sites are colored red on the glycoprotein surfaces. Calculated structure-based evolutionary distances are indicated beside the branches.

We sought to assess whether the functional independence of SosV-RBP from paramyxoviral RBPs with known HN functionality was reflected at a structural level. A soluble construct of SosV-RBP was engineered to include a portion of the N-terminal stalk region (residues 125−157) and the receptor binding β-propeller domain (residues 158−582) (Fig. 2*A*). SosV-RBP was crystallized and the structure was determined to 2.50-Å resolution using the structure of MuV-RBP (PDB ID code 5B2C) (43) as a molecular replacement search model (*SI Appendix*, Table S1).

Two near-identical molecules of SosV-RBP were observed in the asymmetric unit (root-mean-square deviation [RMSD] of 0.5 Å over 366 aligned Cα atoms). Residues ranging from 158 to 582 correspond to the canonical six-bladed β-propeller of the paramyxoviral attachment glycoprotein (25), with each blade (β1 to β6) composed of four antiparallel β-strands (Fig. 2*A*). Residues in the N-terminal stalk region (residues 125−155) and loops β3L23 (344–373) and β5L01 (464–479) were disordered and directed toward solvent channels in the crystal (Fig. 2*A*), suggestive that they may be intrinsically flexible in the absence of neighboring RBP and fusion proteins, as presented on the virion surface.

Consistent with the widely observed role of β-propeller–displayed N-linked glycans in protein folding, virulence, host immune evasion, and activation of host-cell fusion cascades (54–58), the SosV-RBP β-propeller is highly glycosylated, encoding six N-linked glycosylation sequons (NXS/T, where X≠P). Electron density corresponding to *N*-acetylglucosamine moieties were observed at all predicted sequons (Asn201, Asn265, Asn340, Asn403, Asn416, and Asn455) (Fig. 2*A*), suggestive that these sites may be occupied on the native virion.

Overlay analysis reveals that SosV-RBP shares the greatest level of structural conservation with orthoavula-, orthorubula-, and respirovirus HN RBPs (1.7−2.1 Å RMSD upon superposition of equivalent Cα atoms), when compared with protein-binding morbilliviral H RBPs (3.1 Å RMSD upon overlay with measles virus RBP; MV-RBP) and henipaviral G RBPs (2.2−2.3 Å RMSD) (Fig. 2*B*). The relatively close structural correspondence of the SosV-RBP β-propeller scaffold with other paramyxoviral RBPs with HN functionality is also reflected upon structure-based phylogenetic analysis (Fig. 2*C*) (59, 60). Indeed, in line with our previous investigations, which demonstrate that paramyxoviral RBPs structurally classify according to receptor usage (23, 61, 62), the relatively close proximity of SosV-RBP to other RBPs with HN functionality, with respect to henipaviral and morbilliviral RBPs, may reflect that SosV-RBP only recently diverged from sialic acid-binding functionality.

### Unique Dimeric Assembly Supports Sialic Acid-Independent Functionality.

**Fig. 3.**
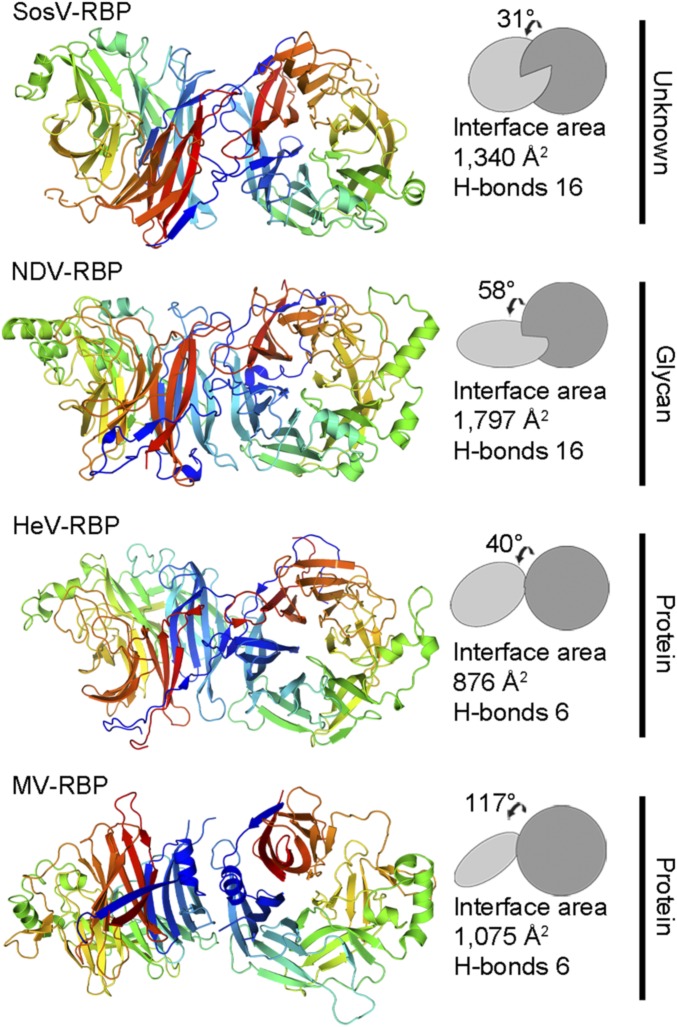
SosV-RBP presents a homodimeric interface that contrasts known RBP dimeric assemblies. Representative paramyxoviral RBP dimers are shown adjacent to their corresponding planes (constructed using UCSF Chimera) (93): SosV; NDV (1E8V) (36); HeV (2X9M) (22); MV (3INB) (67). Interface area (Å^2^) and hydrogen-bonds (H-bonds) were calculated using the PISA server (65). Each structure is annotated with receptor type, if known. Structures are shown in cartoon representation and monomers colored as a rainbow from the N terminus (blue) to the C terminus (red).

Two molecules of SosV-RBP were observed in the asymmetric unit of the crystal and form a putative homodimer through the interaction of the first (β1) and sixth (β6) blades of the β-propeller (Fig. 3). Although we cannot preclude the possibility of preferential crystallization, we note that the formation of such higher-order oligomers has precedent in other dimeric and tetrameric paramyxoviral RBP structures, including NDV-RBP (19, 36), PIV3-RBP (63), PIV5-RBP (18, 20, 64), HeV-RBP (22), and MuV-RBP (43). In addition, the formation of this putative homodimer does not occlude N-linked glycosylation, as expected and consistent with previous analysis of paramyxovirus RBPs (22). The interaction between SosV-RBP protomers occludes ∼1,340 Å^2^ of solvent accessible surface area (as calculated with Proteins, Interfaces, Structures and Assemblies [PISA] European Bioinformatics Institute [EBI]; ref. 65). Furthermore, the interface is stabilized by 16 hydrogen bonds, a number similar to that observed in NDV-RBP, PIV3-RBP, and MuV-RBP structures (average of 14 hydrogen bonds) (Fig. 3).

Interestingly, the interaction area in the SosV-RBP homodimer is less than that observed in NDV-RBP, PIV3-RBP, PIV5-RBP, and MuV-RBP structures (average of ∼1,790 Å), an observation that may be attributed to the absence of contributing contacts from blade one, strand four (β1S4) of the six-bladed β-propeller. Additionally, the angle of association between SosV-RBP protomers (31°) is substantially less than that observed in NDV-RBP (36), PIV3-RBP (63), PIV5-RBP (64), and MuV-RBP (43) (average of 57°) structures. Deviations in association angle have been similarly observed in crystallographically observed HeV-RBP (40°) (22) and MV-RBP (66, 67) (117°) homodimers (Fig. 3). These observations are consistent with the hypothesis that the evolutionary departure of RBPs from sialic acid-binding functionality is accompanied by changes in protomer association angle and interface area to accommodate more bulky, often proteinous receptors (18, 22).

### SosV-RBP Is Structurally Incompatible with Known Modes of Sialic Acid Recognition.

**Fig. 4.**
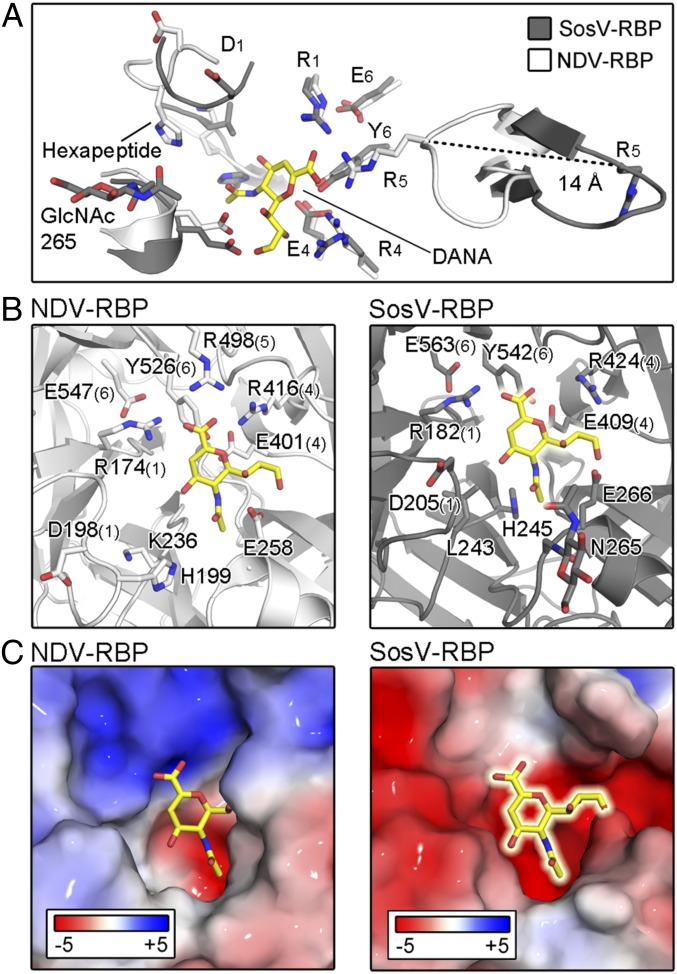
The surface of SosV-RBP is incompatible with known modes of sialic acid recognition. (*A*) Active site of NDV-RBP (white) superposed with SosV-RBP (gray) structure, highlighting the differing locations of the R_5_ residues. Residues are labeled as in Fig. 1. Loops are shown in cartoon representation, with functionally important residues and the inhibitor *N*-Acetyl-2,3-dehydro-2-deoxyneuramic acid (DANA) (yellow) shown as sticks and colored according to atom type. (*B*) Side-by-side representation of the NDV-RBP sialic acid active site bound by DANA (1E8V) and the equivalent site of SosV-RBP with the hypothetical position of DANA shown. While many elements, including the glycerol binding region are structurally conserved, both the hexapeptide motif and triarginyl are structurally different. Structures are represented and colored as above. (*C*) Electrostatic charge differences between NDV-RBP and SosV-RBP surfaces at the NDV-RBP active site. Charges were calculated using the Adaptive Poisson-Boltzmann Solver (95) and are shown in surface representation with residues colored from red to blue (−5 kT·e^−1^ to +5 kT·e^−1^). DANA is represented in both structures as in *B*.

The sialic acid active site of structurally characterized RBPs, including MuV-RBP (43), PIV5-RBP (20), PIV3-RBP (63), and NDV-RBP (36), localizes to a cavity at the top center of the β-propeller fold (Fig. 4*A*). Structural and functional analyses have comprehensively detailed the conserved RBP−glycan interactions facilitated by the seven conserved sialidase residues and hexapeptide motifs, which are essential for HN activity (20, 35–38, 43, 63). In addition to our hemadsorption and sialidase activity analysis (Fig. 1 *B* and *C*), we collected crystallographic data on SosV-RBP crystals soaked with (3-sialyllactose, 30 mM) and cocrystallized in the presence of sialyllactose (3- and 6-sialyllactose, at a 5 times greater molar concentration than protein). However, consistent with our hemadsorption and neuraminidase activity assays (Fig. 1 *B* and *C*), we could find no evidence for glycan binding at the sialic acid cavity (38), nor at the region equivalent to the secondary sialic acid binding site on NDV-RBP (41).

Examination of our unliganded SosV-RBP structure provides a molecular rationale for the absence of this interaction. Indeed, while the glycoprotein presents some features that are conserved with paramyxoviral RBPs with known HN functionality, including a conserved cation binding site (*SI Appendix*, Fig. S2) (36, 63, 64, 68) and residues that would contribute to recognition of the glycerol moeity of sialic acid (Fig. 4 *A* and *B*), the overall configuration of the putative active site is incompatible with known modes of sialic acid recognition. First, we observe that SosV-RBP hexapeptide residues Leu243^SosV-RBP^ and His245^SosV-RBP^ impede into the region where the O_4_ of sialic acid is positioned in other paramyxovirus RBP−sialic acid complex structures (Fig. 4 *A* and *B* and *SI Appendix*, Fig. S3). Second, analysis of the electrostatics in the SosV-RBP cavity reveals dramatically different surface charge properties when compared to paramyxoviral RPBs with HN functionality, where the SosV-RBP presents an extended acidic patch, which is unlikely to be favorable for binding of sialylated glycoconjugates (Fig. 4*C*). Third, the β5L23 loop in SosV-RBP, which encodes Arg514 (R_5_) of the triarginyl motif, does not fold inward, as observed in other sialic acid-binding paramyxoviral RBPs, but peels outwards away from the toroidal axis of the β-propeller toward a small external cavity. This dramatic difference in loop conformation results in a ∼14-Å distance between the equivalent Cα atom of Arg_5_ in SosV-RBP and the typical liganded and unliganded HN RBP (Fig. 4*A* and *SI Appendix*, Fig. S4). We note that the equivalent region in the HN RBPs is structurally conserved and remains unchanged upon ligand recognition (20, 38, 43, 63). Such a large local structural difference in the β-propeller fold may be, in part, attributed to the presence of a disulphide bond in SosV-RBP between residues Cys522 and Cys527 of β5S3 and β5S4 (*SI Appendix*, Fig. S4), respectively, which is not present in other paramyxoviral RBPs with HN functionality, and may contribute to an open conformation of the β5L23 loop (Fig. 4*A*). Combined, the dramatic overall structural and physicochemical differences observed between SosV-RBP and paramyxoviral RBPs with HN functionality is consistent with our hemadsorption and neuraminidase activity analysis (Fig. 1 *B* and *C*), and provides a structural rationale for why the replacement of the hexapeptide motif alone does not result in SosV-RBP obtaining the capability to hydrolyze sialic acid (Fig. 1*C*).

## Discussion

The considerable toll exacted by bat-borne viruses, such as pathogenic henipaviruses, coronaviruses, and filoviruses, upon human health and animal husbandry has provoked worldwide initiatives focused on exploring virus diversity in wildlife and identifying determinants of emergence (69). While a number of sociological, epidemiological, and economic parameters are essential in defining the spillover potential of these emerging and reemerging viruses (70), the ability of virus-displayed glycoproteins to productively attach to and interact with a human host-cell constitutes a fundamental barrier for zoonosis (71, 72). Here, we provide insights into such molecular-level restrictions at the stage of host-cell entry and egress for bat-borne SosV, a recently identified pararubulavirus associated with severe febrile disease.

In line with studies on related pararubulaviruses (7, 44–46), our hemadsorption and sialidase analysis reveals that the absence of the hexapeptide motif in SosV-RBP, which is well conserved in paramyxovirus RBPs with known sialic acid functionality (e.g., MuV-RBP, PIV3-RBP, NDV-RBP, and PIV5-RBP), results in limited hemadsorption and no sialidase activity (Fig. 1). Interestingly, we note that a vaccine strain of MuV has been reported to bind α2,8 sialic acid (73–75). However, this somewhat broadened receptor tropism did not involve a departure from α2,3 and α2,6 specificity, indicative that if SosV-RBP also interacted with this neurotrophic-associated glycan, we would have likely observed hemadsorption and neuraminidase activity in our functional assays (Fig. 1). Similarly, while there was no evidence for sialic acid binding in our crystal soaking and cocrystallization experiments, given that trace hemadsorption activity was observed with respect to an NDV-RBP control (Fig. 1*B*), we cannot discount the possibility of low-affinity interactions with sialic acid. Indeed, such interactions have been shown to augment MERS-CoV infection (76).

Although we were unable to produce a full-length eSosV-RBP construct that includes the four hexapeptide residues missing from SosV-RBP in sufficient yield to assess the effect of these residues on hemadsorption, solubly produced eSosV-RBP exhibited no measurable neuraminidase activity (Fig. 1 and *SI Appendix*, Fig. S1). We rationalize why introduction of the hexapeptide motif is not sufficient to confer hydrolysis activity by showing that SosV-RBP is structurally and physicochemically incompatible with the established mode of paramyxovirus HN-glycan recognition (Fig. 4). Specifically, we find that the region corresponding to the sialic acid binding site on SosV-RBP presents less favorable surface charge properties (Fig. 4*C*), is partially occluded by N-linked glycosylation presented by Asn265, and is sterically disrupted by local structural elements (Fig. 4 *A* and *B*), including the protruding residues, Leu243 and His245 (*SI Appendix*, Fig. S3).

Assuming that an ancestral precursor to SosV utilized sialic acid as a receptor, it seems plausible that the observed structural differences at the sialic acid recognition site may have arisen following the acquisition of binding motifs to a unique receptor (e.g., either protein or glycan specific). Alternatively, given the structural plasticity of the β-propeller (25), it is possible that another site on SosV-RBP may be utilized for receptor recognition, and structural diversification at the original sialic acid binding site may have occurred due to the absence of functional constraints to maintain efficient sialic acid recognition capacity. Furthermore, we note that the mode of SosV-RBP homodimerization deviates from that the conserved ∼60° association angle observed in sialic acid-specific MuV-RBP, hPIV5-RBP, PIV3-RBP, and NDV-RBP structures, a feature in common with protein-binding HeV-RBP and MV-RBP glycoproteins, and supportive of the hypothesis that the acquisition of new receptor-binding modularity may require alteration to the higher-order attachment glycoprotein assembly (18, 22).

Interestingly and consistent with genetic analysis (8), structure overlay of available paramyxoviral attachment glycoprotein structures reveals that the overall six-bladed β-propeller fold of SosV-RBP more closely matches sialic acid-binding RBPs than henipaviral or morbilliviral RBPs (Fig. 2*B*). Combined with the observation that SosV-RBP does not appear to share structural features required for ephrin or SLAMF1/nectin-4 recognition (Fig. 2*B*), we suggest that SosV may have more recently diverged from a common sialic acid-specific ancestral paramyxovirus than known protein-specific henipa- and morbilliviruses (Fig. 2*C*). Moreover, this analysis demonstrates the smallest known level of structural reorganization to the β-propeller scaffold required for sialic acid-independent paramyxovirus host-cell attachment.

The burden of newly emerging viruses in humans may be underestimated. For example, a recent study in Uganda reported that 62% of cases of severe febrile illness were misdiagnosed as malaria due to resource limitations in clinics (77). While the potential biomedical and economic impact of pararubulavirus emergence remains to be fully established, several pararubulaviruses, in addition to SosV, have shown the ability to cross the species barrier and cause disease. For example, MenV infects both pigs, fruit bats, and humans (45, 78), and laboratory models of the bat-borne Achimota viruses (15) have been found to cause respiratory disease in ferrets.

Our integrated structural and functional investigation, combined with studies supporting the sialic acid independence of Menangle virus (MenV), Teviot virus (TevPV), and Tioman virus (TioV) (7), indicate that pararubulavirus RBPs are functionally distinct from characterized paramyxoviral H, HN, and G RBPs, and undergo a novel host cell entry pathway. While the receptor(s) utilized by SosV remains unknown, this work broadens our appreciation of the diverse host receptors utilized by paramyxoviruses. Future efforts to characterize the receptor(s) utilized by this group of emerging pathogens will be essential for understanding cellular, tissue, and species tropism characteristics, as well as rationalizing the spillover potential of these emerging viruses.

## Materials and Methods

### Protein Production.

SosV-RBP (GenBank accession no. NC_025343.1) cDNA was synthesized by GeneArt LTD. SosV-RBP (residues 125−582) was cloned into the pURD vector alongside a 3C protease cleavable N-terminal SUMO tag and hexahistidine tag (79) and used to generate a stable HEK293T cell line (80). When required for crystallization, upscaling of stably expressing cells was performed in the presence of kifunensine (81). All proteins were purified using immobilized metal-affinity chromatography (IMAC) and subsequently N-linked glycans were cleaved at the di-*N*-acetylchitebiose core using endoglycosidase F1 (EndoF1) (10 µg/mg protein, 12 h, 21 °C). Size-exclusion chromatography (SEC) in 150 mM NaCl, 10 mM Tris pH 8.0, buffer using a Superdex 200 10/30 column was performed. When required for crystallography, N-terminal SUMO tag and hexahistidine tag cleavage was performed (1:10 molar ratio of protein to 3C protease, 12 h, 21 °C), prior to IMAC (to separate tag from cleaved protein) and SEC.

### Crystallization and Structure Determination.

SosV-RBP crystals were grown using nanoliter-scale sitting-drop vapor diffusion at room temperature, using 100 nL of protein and 100 nL of reservoir (82). Crystals grew after 25 d in a precipitant containing 0.2 M magnesium acetate tetrahydrate, 0.1 M sodium cacodylate pH 6.5, 20% PEG 8000. Crystals were immersed in 20% glycerol prior to cryo-cooling by plunging into liquid nitrogen.

Data collection was performed at wavelength 0.9795 Å at beamline I04, Diamond Light Source (DLS), United Kingdom. Images were integrated and scaled using the XIA2 pipeline (83). SosV-RBP was solved with MuV-HN (PDB ID code 5B2C) as the search model (43), using Phaser within the PHENIX suit (84, 85). Model building and structure refinement were iteratively performed with COOT and Phenix.Refine, respectively (86, 87). Noncrystallographic symmetry restraints were employed throughout, and Translation-libration-screw parameters were employed for later rounds of refinement. Structures were validated with Molprobity (88). Crystallographic data processing and refinement statistics are presented in *SI Appendix*, Table S1. The atomic coordinates and structure factors of SosV-RBP were deposited in the Protein Data Bank (PDB), PDB code 6SG8 (89).

### Hemadsorption Assay.

The hemadsorption method to determine sialic acid binding was adapted from that developed by Morrison and McGinnes (47). Briefly, full-length SosV-RBP (residues 1−582) and NDV-RBP (residues 1−577, GenBank accession no. AF212323.1) were cloned into the pHLsec vector with a C-terminal hexa-histidine tag (90), and transfected with Lipofectamine 2000 (ThermoFisher, product no. 11668030) into HEK 293T cells. HEK 293T cell monolayers were washed with phosphate buffer saline (PBS), pH 7.4 (with MgCl_2_ and CaCl_2_), 24 h following transfection, prior to incubation with 2% sheep blood (Thermo Scientific Oxoid, 12967755) at 4 °C for 30 min. Cells were gently washed to remove unadsorbed erythrocytes, prior to lysing absorbed erythrocytes using 50 mM Tris, pH 7.4, 5 mM EDTA (ethylenediaminetetraacetic acid), 150 mM NaCl and 0.5% Nonidet P-40. Absorbance at 540 nm was measured using a CLARIOStar plate reader (BMG Labtech).

### Neuraminidase Assay.

Neuraminidase activity was determined through hydrolysis of the substrate 2′-(4-methylumbelliferyl)-α-d-*N*-acetylneuraminic acid (MU-Neu5Ac; Sigma-Aldrich, product no. M8639), as described previously (38, 48). The full-length SosV-RBP and NDV-RBP constructs presented above were transfected with Lipofectamine 2000 into HEK 293T cells. Twenty-four hours after transfection, HEK 293T monolayers were washed with PBS, pH 7.4, counted and seeded in a 96-well black nontransparent plate at a density of 25,000 cells per mL. Cells were pelleted by spinning at 1,500 rpm for 5 min, and supernatant was replaced with 0.1 M sodium acetate, pH 6.0 containing 1 mM MU-Neu5Ac. The plate was incubated for 1 h at 37 °C prior to addition of 0.25 M glycine buffer, pH 10.7 to stop the reaction. The amount of free 4-methylumbelliferone was fluorimetrically determined at 365 nm for excitation and 450 nm for emission using a CLARIOStar plate reader (BMG Labtech).

### Cell Surface Expression.

ELISA (enzyme-linked immunosorbent assay) was utilized to measure expression of full-length SosV-RBP and NDV-RBP on HEK293T cells (91). Lipofectamine-2000 transfected cells were washed with PBS pH 7.4 following 18 h incubation at 37 °C, 5% CO_2_, counted and seeded into an ELISA plate at a density of 25,000 cells per mL. Cells were bound overnight at 4 °C prior to fixation for 15 min in 4% paraformaldehyde. Following thorough washing, cells were blocked in PBS-5% milk for 1 h and subsequently reacted with rabbit anti-6xhis-tag antibody (Abcam, product no. ab9108) for 1 h at room temperature. Cells were repeatedly washed prior to addition of horseradish peroxidase-conjugated goat anti-rabbit IgG (Vector Laboratories, product no. PI-1000), for 1 h at 21 °C. Cells were repeatedly washed prior to addition of TMB substrate kit (Pierce). Stop solution (2 M sulphuric acid) was added following a 15-min incubation at room temperature, and absorbance was read at 430 nm using a using an Infinite F200 plate reader (TECAN).

### Production of the Hexapeptide Motif eSosV-RBP Mutant.

The splice-by-overlap extension PCR method was utilized to generate a soluble contruct of SosV-RBP (eSosV-RBP, residues 125−582) bearing the following hexapeptide motif site-directed substitutions: R242N, L243R, K244Y, and H245S (Fig. 1*A*). eSosV-RBP was cloned into a pHLsec vector containing an N-terminal SUMO tag and hexahistidine tag (79).

### Warren Method for Determining Free Sialic Acid.

Alongside a mock-transfected negative control, HEK 293T cell monolayers were transiently transfected using Lipofectamine 2000 with eSosV-RBP, WT SosV-RBP, and NDV-RBP (residues 47−570) similarly cloned into a pHLsec vector encoding an N-terminal SUMO tag and hexahistidine tag. An ELISA plate was incubated overnight at 4 °C with mouse-derived monoclonal anti-penta-histidine antibody (Qiagen, product no. 34660) diluted 1:200 in PBS, pH 7.4 (Gibco). Plates were washed and stained with HEK293T cell supernatants containing soluble NDV-RBP, WT SosV-RBP, eSosV-RBP, and the mock-transfected control. Neuraminidase activity was assayed by measuring levels of free sialic acid (FSA) following incubation with 50 µM fetuin (Sigma Aldrich, product no. F3004) for 18 h at 37 °C in PBS, pH 7.4 (Gibco). Using a sialic acid assay kit (Sigma Aldrich, product no. MAK314) based on the Warren method for assaying sialic acid (49), FSA was oxidized to formylpyruvic acid and subsequently reacted with thiobarituric acid to form a pink product, which was fluorimetrically measured (λex = 555/λem = 585 nm) using a CLARIOStar plate reader (BMG Labtech).

### Structural Phylogenetic Analysis.

Structural phylogenetic analysis was performed with the Structural Homology Program (SHP) (59, 60) using paramyxoviral RBP monomers. The resulting evolutionary distance matrix was used to construct an unrooted phylogenetic tree with the PHYLogeny Inference Package (PHYLIP) (92).

### Dimer Angle Analysis.

Analysis of relative angles monomers within the paramyxoviral dimers was performed with UCSF Chimera (93). To calculate the angle between the monomers of a dimer, planes representing the top faces of the monomers were constructed based upon conserved stretches of paramyxoviral RBP sequence, using the “Define plane functionality.”

## Supplementary Material

Supplementary File
