# Supplementary material for: A structure-based rationale for sialic acid independent host-cell entry of Sosuga virus
Source: Proc Natl Acad Sci U S A. 2019 Oct 7;116(43):21514–20. doi: 10.1073/pnas.1906717116 (PMC6815108; doi:10.1073/pnas.1906717116)
Supplement: Supplementary File [file pnas.1906717116.sapp.pdf]

PNAS
www.pnas.org

Supplementary Information for

A structure-based rationale for sialic acid independent host-cell entry of Sosuga virus.

Alice J. Stelfox and Thomas A. Bowden

Thomas A. Bowden
Email: Thomas.Bowden@strubi.ox.ac.uk

This PDF file includes:

Figures S1 to S4

Table S1

SI References

www.pnas.org/cgi/doi/10.1073/pnas.1906717116

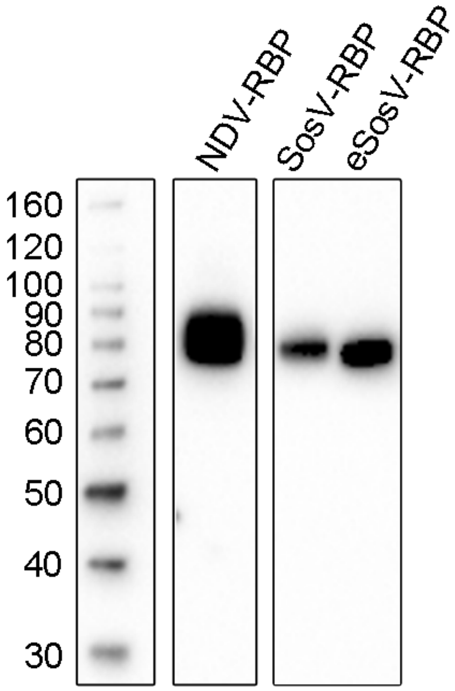

Supplementary Fig. S1. Western blot demonstrating soluble expression of correctly folded RBPs. HEK293T cells were transiently transfected with NDV-RBP, wildtype SosV-RBP, and eSosV-RBP. All constructs encoded an N-terminal hexa-histidine tag and SUMO-tag. The expression of folded soluble protein harvested from the supernatants of transfected HEK293T cells was determined using western blotting with a mouse- derived monoclonal anti-penta-histidine antibody. Samples were run alongside Benchmark™ Protein Ladder (Thermo, left), on the same gel with the same exposure time.

Supplementary Table S1. Crystallographic data collection and refinement statistics.

	SosV-RBP
**Data collection statistics**	
**Beamline**	DLS i04
**Spacegroup**	$P2_{(1)}$
**Cell dimensions**	
**a, b, c (Å)**	75.7, 84.0, 81.7
**α, β, γ (°)**	90.0, 112.9, 90.0
**Resolution range (Å)**	51.60–2.50 (2.54–2.50)[a]
**Wavelength (Å)**	0.9795
**R_{merge}**	0.075 (0.941)
**R_{meas}**	0.081 (1.021)
**R_{pim}**	0.031 (0.392)
**I/σ I**	13.5 (1.5)
**$CC_{1/2}$**	1.00 (0.80)
**Completeness (%)**	99.8 (98.9)
**Multiplicity**	6.7 (6.5)
**Refinement statistics**	
**Resolution (Å)**	43.44-2.50 (2.59–2.50)
**No. reflections**	32,699
**R_{work} / R_{free}[b] (%)**	19.5/23.3
**No. atoms**	
**Protein**	5,870
**Ligand/ion**	182
**Water**	39
***B*-factors**	
**Protein**	77.6
**Ligand**	91.2
**Water**	61.0
**R.m.s deviations[c]**	
**Bond lengths (Å)**	0.003
**Bond angles (°)**	0.65
**Ramachandran analysis[d]**	
**Residues in favoured region (%)**	96.61
**Residues in allowed region (%)**	3.26

[a]Numbers in parentheses refer to the relevant outer resolution shell.
[b]R_{free} is calculated as for R_{work}, but using only 5% of the data which were separated prior to refinement.
[c]r.m.s deviations: root mean square deviation from ideal geometry.
[d]Determined using the Molprobity server [1].

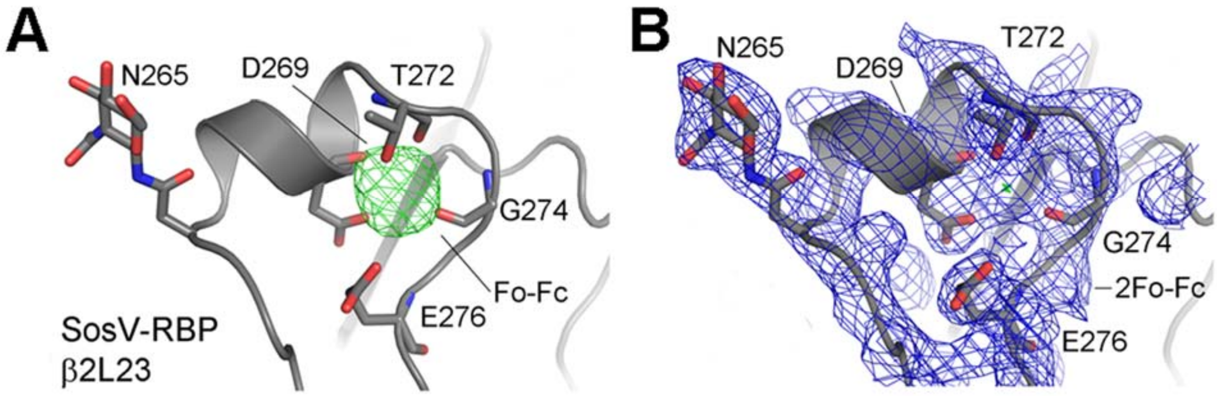

Supplementary Fig. S2. The SosV-RBP loop β2L23 coordinates a putative calcium cation. (a) The *Fo–Fc* difference map indicates the presence of a cation coordinated by the SosV-RBP loop β2L23 via the main chain carbonyl oxygen of residues Asp269, Thr272, and Gly274, and the side chains of Asp269, Thr272, and Glu276. A similar mechanism of stabilization of β2L23 through coordination of a calcium cation has been observed in several HN RBP structures, and loss of calcium inhibits activity in NDV-RBP [2]. The *Fo–Fc* difference map is rendered at 2.0 σ and represented as mesh (green). **(b)** Fitting of a calcium ion (green) into loop β2L23 is consistent with the 2*Fo–Fc* difference map. The 2*Fo–Fc* electron density is rendered at 2.0 σ and displayed as mesh (blue). SosV-RBP is displayed in cartoon (gray) representation, with important residues and glycans displayed as sticks.

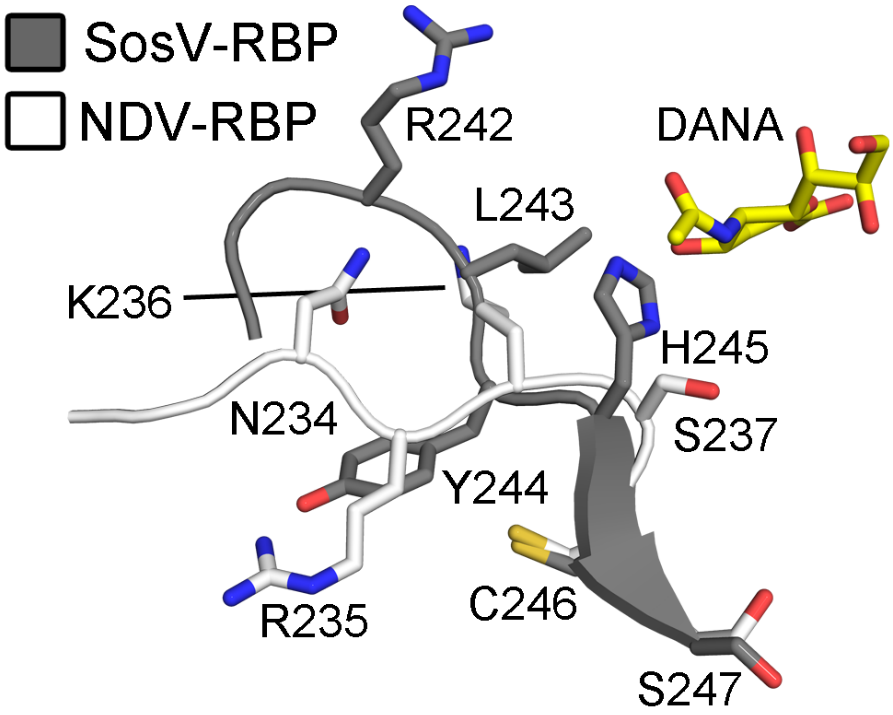

Supplementary Fig. S3. The SosV-RBP hexapeptide motif region is structurally distinct to NDV-RBP and other HN-RBPs. SosV-RBP (gray) and NDV-RBP (white) (1E8V) [3] are shown in cartoon representation with the relevant side chains represented as sticks. N-Acetyl-2,3-dehydro-2-deoxyneuramic acid (DANA) is shown as yellow sticks. Homologous residues are numbered according to SosV-RBP. In contrast to NDV-RBP, SosV-RBP residues Arg242, Leu243, and His245 impede into the putative sialic acid binding site. The side-chain of Lys236 in NDV-RBP has been observed to adopt different conformations between ligand-free and ligand-bound states [2], however, subsequent structural studies of other HN RBPs commonly show the above orientation [4-7].

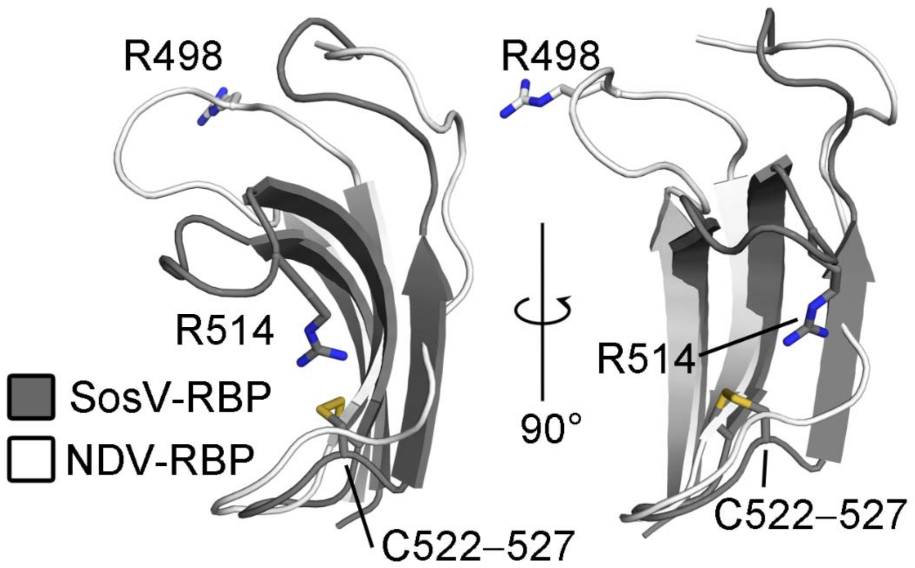

Supplementary Fig. S4. The unique disulfide formed between Cys522 and Cys527 could contribute to the unique orientation of loop β5L23. Both SosV-RBP (gray) and NDV-RBP (white) (1E8V) are shown in cartoon representation with the relevant residues represented using sticks and colored according to atom. The conserved triarginyl motif of the SosV-RBP structure is disrupted, loop β5L23 bends outwards, away from the putative active site. The disulfide bond (Cys522–Cys527) formed between β5S3 and β5S4 has not been observed in other paramyxovirus HN RBPs and may contribute to the structural displacement of residue Arg514 in SosV-RBP.

Supplementary references

1. Chen, V.B., et al., *MolProbity: all-atom structure validation for macromolecular crystallography.* Acta crystallographica. Section D, Biological crystallography, 2010. **66**(Pt 1): p. 12-21.
2. Connaris, H., et al., *Probing the sialic acid binding site of the hemagglutinin-neuraminidase of Newcastle disease virus: identification of key amino acids involved in cell binding, catalysis, and fusion.* J Virol, 2002. **76**(4): p. 1816-24.
3. Crennell, S., et al., *Crystal structure of the multifunctional paramyxovirus hemagglutinin-neuraminidase.* Nature Structural Biology, 2000. **7**(11): p. 1068-1074.
4. Kubota, M., et al., *Trisaccharide containing alpha 2,3-linked sialic acid is a receptor for mumps virus.* Proceedings of the National Academy of Sciences of the United States of America, 2016. **113**(41): p. 11579-11584.
5. Lawrence, M.C., et al., *Structure of the haemagglutinin-neuraminidase from human parainfluenza virus type III.* J. Mol. Biol., 2004. **335**(5): p. 1343-1357.
6. Welch, B.D., et al., *Structure of the parainfluenza virus 5 (PIV5) hemagglutinin-neuraminidase (HN) ectodomain.* PLoS Pathog, 2013. **9**(8): p. e1003534.
7. Yuan, P., et al., *Structural studies of the parainfluenza virus 5 hemagglutinin-neuraminidase tetramer in complex with its receptor, sialyllactose.* Structure, 2005. **13**(5): p. 803-15.
